# Effects of soft robotic exoskeleton for gait training on clinical and biomechanical gait outcomes in patients with sub-acute stroke: a randomized controlled pilot study

**DOI:** 10.3389/fneur.2023.1296102

**Published:** 2023-11-02

**Authors:** Ruimou Xie, Yanlin Zhang, Hainan Jin, Fei Yang, Yutong Feng, Yu Pan

**Affiliations:** ^1^Department of Rehabilitation Medicine, Beijing Tsinghua Changgung Hospital, Beijing, China; ^2^School of Clinical Medicine, Tsinghua University, Beijing, China

**Keywords:** stroke rehabilitation, soft exoskeleton, exoskeleton robotics, gait training, walking function

## Abstract

**Background:**

Ankle function impairment is a critical factor impairing normal walking in survivors of stroke. The soft robotic exoskeleton (SRE) is a novel, portable, lightweight assistive device with promising therapeutic potential for gait recovery during post-stroke rehabilitation. However, whether long-term SRE-assisted walking training influences walking function and gait quality in patients following subacute stroke is unknown. Therefore, the primary objective of this study was to assess the therapeutic effects of SRE-assisted walking training on clinical and biomechanical gait outcomes in the rehabilitation of patients with subacute stroke.

**Methods:**

A group patients who had experienced subacute stroke received conventional rehabilitation (CR) training combined with 10-session SRE-assisted overground walking training (30 min per session, 5 sessions/week, 2 weeks) (SRE group, *n* = 15) compared with the control group that received CR training only (CR group, *n* = 15). Clinical assessments and biomechanical gait quality measures were performed pre-and post-10-session intervention, with the 10-Minute Walk Test (10MWT) and 6-Minute Walk Test (6MWT) used to define the primary clinical outcome measures and the Functional Ambulation Category, Fugl-Meyer Assessment for Lower Extremity (FMA-LE) subscale, and Berg Balance Scale defined the secondary outcome measures. The gait quality outcome measures included spatiotemporal and symmetrical parameters during walking.

**Results:**

After the 10-session intervention, the SRE and CR groups exhibited significant within-group improvements in all clinical outcome measures (*p* < 0.05). Between-comparison using covariance analyses demonstrated that the SRE group showed greater improvement in walking speed during the 10MWT (*p* < 0.01), distance walked during the 6MWT (*p* < 0.05), and FMA-LE scores (*p* < 0.05). Gait analyses showed that the SRE group exhibited significantly improved spatiotemporal symmetry (*p* < 0.001) after 10-session training, with no significant changes observed in the CR group.

**Conclusion:**

Compared with CR training, SRE-assisted walking training led to greater improvements in walking speed, endurance, and motor recovery. Our findings provide preliminary evidence that SRE may be considered for inclusion in intensive gait training clinical rehabilitation programs to further improve walking function in patients who have experienced stroke.

## 1. Introduction

Stroke is the leading factor of severe disability, and frequently leads to impaired walking function (1–3). Approximately 60% of patients with stroke experience improvement in gait function within the initial months after stroke; however, over one-third of patients continue to experience difficulties with independent ambulation (4, 5). Therefore, improving gait function is vital for patients who have experienced stroke and is the primary goal of rehabilitation protocols (6).

Hemiparetic ankle deficits are a major cause of impaired gait function after stroke (7–9). In patients with healthy gait, both lower extremities generate ankle dorsiflexion torque to promote foot clearance and ankle plantarflexion torque required for the propulsion for body movements (10). However, in patients with a stroke episode, impaired ankle dorsiflexion and plantarflexion caused by hemiparesis impairs both ground clearance and propulsion (11–13). Therefore, individuals with hemiparesis often adopt compensatory gait patterns, like vaulting gait and circumduction, to overcome the insufficiency in foot clearance and propulsion (14–16). These asymmetrical gait patterns impair walking ability, resulting in reduced walking speed (17) and endurance (18) and increased energy consumption (19).

The soft robotic exoskeleton (SRE) is a portable, lightweight, and wearable walking-assistive device that improves gait function and mobility in patients with stroke by facilitating normal movement in the paretic ankle during walking (20, 21). Previous studies have shown that the use of SRE immediately and positively improves gait function in patients with stroke. For example, initial proof-of-concept studies reported that assistance from the SRE may contribute to immediate enhancements in kinematics, kinetics, walking speed, and endurance of post-stroke walking (20–22). In addition, recent clinical exploratory studies have shown that the promising effects of SRE on ambulation function could be extended to unassisted gait after the patient has removed the devices (the therapeutic effects) (23, 24). A recent multi-center clinical study evaluated the effects of a commercially available SRE in survivors of stroke and demonstrated that SRE-augmented gait rehabilitation could improve the maximum walking velocity after five training sessions (24). Another recent SRE-augmented locomotion study conducted a high-intensity walking training with SRE and reported enhancements in clinical and biomechanical outcomes in five survivors of chronic stroke after 18 training sessions (23). These studies have provided preliminary evidence that SRE has immediate gait restorative effects and therapeutic potential for long-term gait recovery in survivors of chronic stroke.

To the best of our knowledge, no randomized controlled trial (RCT) has been conducted to evaluate the therapeutic effects of SRE when integrated with conventional rehabilitation (CR) protocols on the recovery of walking function in patients who have experienced stroke; furthermore, none have explicitly focused on individuals with hemiplegia during the subacute post-stroke phase. According to previous reviews of electromechanically assisted gait training, future research and clinical practice on robotics should focus on patients in the sub-acute post-stroke phase as the neurological recuperation typically occurs within the initial 6-week period after stroke (25).

The objective of this pilot study was to assess the therapeutic effects of SRE-assisted walking training on clinical and biomechanical gait outcomes in patients with subacute stroke. It was hypothesized that CR training combined with SRE-assisted walking training would be more effective in enhancing walking functional recovery in these patients than CR training alone.

## 2. Methods

### 2.1. Participants

This single-site, parallel-group, sham-controlled RCT was conducted in Beijing between May 2022 and April 2023. Survivors of subacute stroke with mild motor impairment in the paretic ankle were screened and recruited from the Beijing Tsinghua Changgunng Hospital (Beijing, China). Inclusion criteria were: (i) 18 years < age < 80 years, (ii) one-sided ischemic or hemorrhagic stroke, (iii) 2 weeks < post-stoke <24 weeks, (iv) ability for ambulation with less or no assistance (Functional Ambulation Category [FAC] ≥ 3) for at least 2 min, (v) ability to carry out a 3-step command and communicate basic needs, (vi) intact skin on which the device is connected to the participants, (vii) Modified Ashworth Scale score for muscle tone at ≤2 in lower limbs, and (viii) sufficient passive joint range of motion for safe ambulation.

Exclusion criteria were: (i) significant musculoskeletal or other neurological conditions affecting mobility, (ii) severe comorbidities impairing ability to participate, (iii) severe aphasia, (iv) severe peripheral artery disease, (v) uncontrolled hypotension/hypertension, (vi) recruitment in other clinical trial, and (vii) open wounds or broken skin at the lower extremities.

### 2.2. Sample size and randomization

The sample size of the present study was estimated based on one previous study that investigated the effect of the robotic ankle exoskeleton on gait recovery of in stroke survivors, with a sample size was 19 for two groups (17). Considering the shedding and elimination (30%), the estimated sample size for the current study was set as 30 for the two groups (*n* = 15 each).

After baseline testing, stroke survivors were randomly assigned in a 1:1 ratio into either the SRE or CR group using computer-generated simple random tables. The sequence tables were stored using a closed envelope by an investigator who did not participate in the training and assessment. On registration of a newly eligible participant, an enveloped was randomly extracted, which then informed the therapist of the group allocation. Pre-and post-intervention assessments were performed by the same certified therapists blinded to the group allocation. This research was approved by the ethics committee of Beijing Tsinghua Changgung Hospital. All participants signed an informed consent in accordance with the Declaration of Helsinki (Clinical Trial Registry: ChiCTR2300068869).

### 2.3. Soft robotic exoskeleton

**Figure 1:**
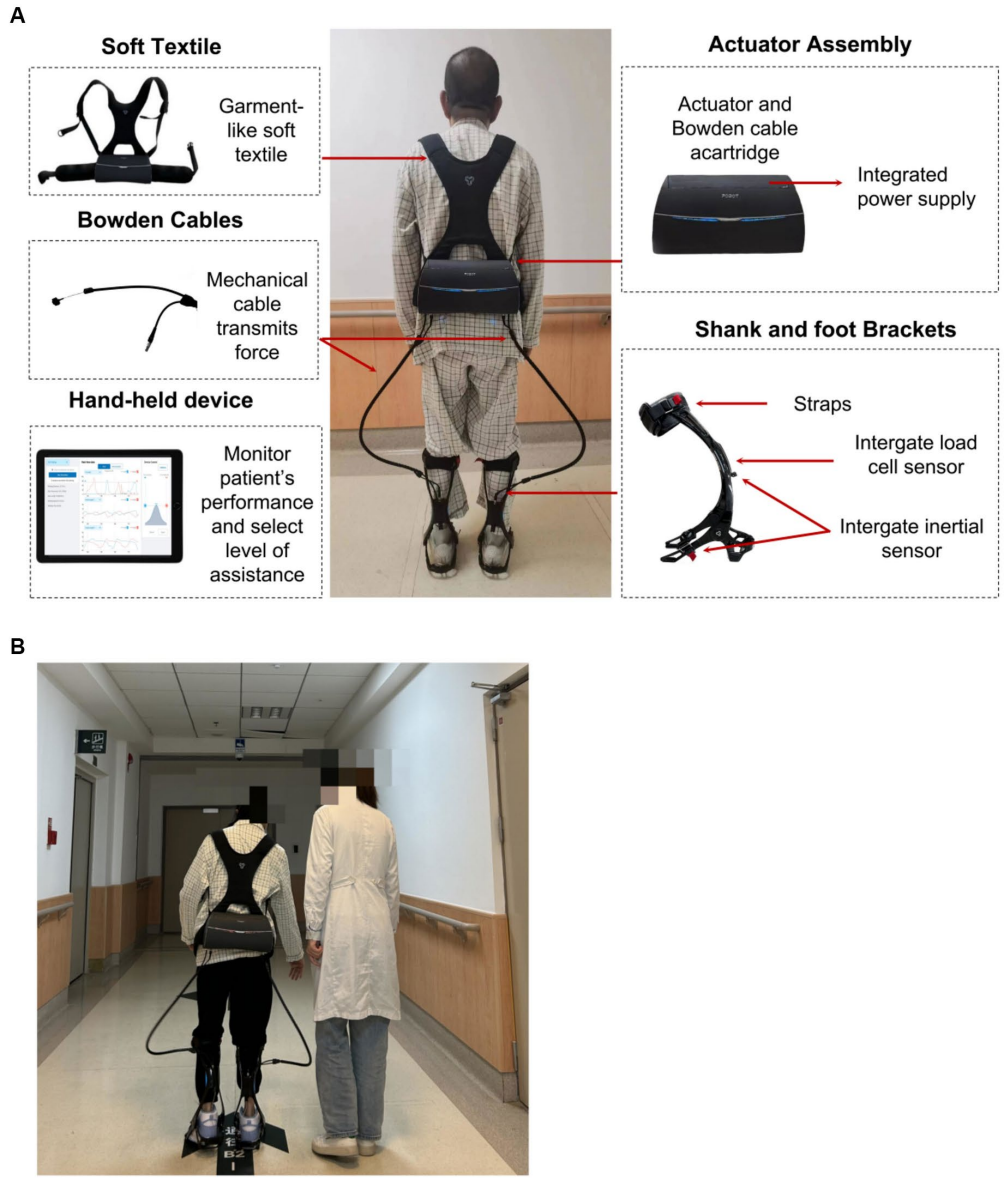
**(A)** Yrobot Relink™ soft robotic exoskeleton, **(B)** SRE-assisted overground walking training.

This study utilized the Yrobot Relink^™^ (Yrobot Inc., Suzhou, China), a commercially available SRE for individuals diagnosed with stroke for overground walking. The Yrobot Relink™ was developed based on the soft-exosuit technique (26) and uses a carbon fiber shank and foot brackets instead of the original calf warp made of flexible fabric; this enhancement provides better mechanical support for the ankle and lower leg during loaded walking. This device comprises of garment-like soft textiles, a wearable actuator assembly with an integrated power supply, Bowden cables, two carbon fiber shank and foot brackets, and wearable sensors (Figure 1). The two Bowden cables span the left and right legs to transmit assistive forces generated by the actuator to the ankles; when the cable is retracted, an ankle plantarflexion torque is produced.

Inertial sensors attached to the shank and foot brackets can collect kinematic data of the wearer’s leg and foot (e.g., displacement and orientation of shank and foot, ankle angle, and spatio-temporal gait parameters). Load cell sensors are strategically positioned at the end of each cable to monitor the user-exoskeleton interaction, ensuring attainment of the desired assisstance level. The amalgamation of inertial sensor and load cell data allow for the accurate identification of gait events and the timing of active ankle plantarflexion assistance, and the assistance force profile of the exoskeleton is adaptively adjusted to match the voluntary movement of the wearer. The force profile of plantarflexion assistance was close to the first half-time period of a sinusoid wave across the terminal stance and pre-swing phases. The sinusoidal motion initiates posterior to mid-stance, with maximum assistance occurring near (usually posterior to) the heel-off moment. The maximum assistance level was controlled by a trained physical therapist. Additionally, the Yrobot Relink™ exoskeleton possesses the capability to adapt and tailor its assistance by learning and accommodating the unique gait characteristics of each user, aligning its assistance with their natural gait.

The total weight of the device is about 3.3 kg, with most of its weight situated closely within the waist-worn backpack. Revolute joints that restrict ankle inversion without limiting dorsiflexion and plantarflexion could be utilized for users requiring mediolateral ankle support in addition to ankle plantarflexion and dorsiflexion assistance. The built-in spiral torsion spring of the revolute joints prevented foot drops during the swing phase. Inertial sensors attached to the shank and foot brackets measured gait events and automated the timing of active ankle plantarflexion assistance. Load cell sensors placed at the extremity of each cable were employed to monitor the user-exoskeleton interaction and to ensure the attainment of the desired assistance level. Therapists were able to monitor the users’ performance and to choose and advance the level of aid in real-time by utilizing a handheld device featuring a graphical interface.

### 2.4. Interventions

All recruited participants underwent CR training (physiotherapy and occupational therapy) in an inpatient rehabilitation center, including standard upper and lower extremity training for strength, transfer, balance, stepping, and activity of daily living. Each session was 60 min and was conducted 5 times weekly for 2 weeks, totaling 10 training sessions.

For participants assigned to the SRE group, 30-min SRE-assisted training (5 sessions/week, 10 sessions) was integrated into their CR protocol (60 min/session, 5 sessions/week) without time compensation. In each training session, participants completed 30-min SRE-assisted overground walking along a 20-m circular walkway at a comfortable, self-selected speed. In the first session, an additional 10 min of pretraining was provided to the participants for fitting and familiarization with the SRE. Notably, 25% of the user’s body weight was used as the target level for bilateral plantarflexion assistance during walking training (20, 22). Participants were allowed rest breaks as needed; however, the resting time was also counted as training time. All physiotherapy sessions were conducted by two licensed physiotherapists with >10 years of clinical practice in stroke rehabilitation. Physiotherapists were trained in intervention protocols in advance. During each training session, the physiotherapists walked beside the paretic sides of the participants to ensure safety. They evaluated vital signs and skin integrity at the beginning and end of each session. Walking distance and steps were covered, and adverse events were documented in each session.

### 2.5. Outcome measures

The primary outcomes in the present study included the self-selected walking speed during the 10MWT and the distance walked during the 6MWT. In the 10MWT, participants were directed to walk at a self-selected speed on a 10-metre path, and the total walking time was recorded. In the 6MWT, participants were instructed to walk back and forth along a 30-meter path at a self-selected, comfortable speed, with the aim of covering as much distance as possible within a fixed 6 min-time frame; the walking distance was subsequently measured. A 6-h rest period was incorporated between the 6MWT and the 10MWT to mitigate the potential impact of fatigue on the test results.

Previous studies have shown that walking speed and endurance are closely associated with the level of functional independence (27, 28) and are the primary goals of rehabilitation intervention after stroke (29, 30). Secondary outcome measures included the FAC, Berg Balance Scale (BBS), and Fugl-Meyer Assessment for lower limb subscale (FAM-LE). All clinical outcomes were assessed by blinded assessors at two timepoints: before training (pre-intervention) and after 10 training sessions (post-intervention). The same assessor conducted both pre-and post-assessments for each participant. All clinical outcomes were assessed in participants who did not wear SRE or orthotics.

To further explain the changes in gait performance, gait quality was measured to supplement the clinical assessment outcomes. Spatiotemporal and symmetry parameters during walking without assistance were collected at two timepoints (pre-and post-intervention) using an instrumented walkway (ProtoKinetics, Havertown, PA, United States).

In the trial session, the patients were required to walk at a self-selected speed on an instrumented walkway repeatedly until sample data from six walking cycles were successfully collected. Spatiotemporal parameters included step length, swing time, and stance time of both unaffected and affected sides. The Temporal Symmetry Ratio (TSR) and Spatial Symmetry Ratio (SSR) metrics were used to evaluate gait symmetry:


(1) $$\begin{array}{l} \mathrm{Temporal}\;\mathrm{swing}\;\mathrm{stance}\;\\ \mathrm{symmetry}\;\left(\mathrm{TSSS}\right)=\left(\mathrm{stepswing}\;\mathrm{time}\right)/\left(\mathrm{step}\;\mathrm{stance}\;\mathrm{time}\right) \end{array}$$

(2) $$TSR=\left(\frac{\max\;\left(\mathrm{affected}\;\mathrm{TSSS},\mathrm{unaffected}\;\mathrm{TSSS}\right)}{\min\;\left(\mathrm{affected}\;\mathrm{TSSS},\mathrm{unaffected}\;\mathrm{TSSS}\right)}\right)$$

(3) $$SSR=\left(\frac{\max\;\left(\mathrm{affected}\;\mathrm{step}\;\mathrm{length},\mathrm{unaffected}\;\mathrm{step}\;\mathrm{length}\right)}{\min\;\left(\mathrm{affected}\;\mathrm{step}\;\mathrm{length},\mathrm{unaffected}\;\mathrm{step}\;\mathrm{length}\right)}\right)$$

where the step swing time, stance time, and length of each limb were presented as the mean of six gait cycles (31). To improve the accuracy of the statistical analyses, the numerators of Eqs 1, 2 used the maximum value from either the affected or unaffected side data, whereas the denominators used the minimum value. This approach ensured that the results were not skewed by values <1.0 (32). TSR and SSR of 1 were defined as having perfect symmetry between the limbs (32).

In addition, exploratory correlation analyses between SRE-induced changes in spatiotemporal symmetry ratios and changes in primary clinical outcomes (gait speed and walking distance) were performed to investigate any potential mechanisms underlying SRE-induced changes in walking performance.

### 2.6. Statistical and minimal important difference analyses

Data were analyzed using IBM SPSS v. 25 (SPSS Inc., Chicago, IL, United States). Continuous variables are presented as mean [standard deviation, (SD)], ordinal variables as median [interquartile range (IQR)], and categorical variables as counts (percentages). All outcomes were analyzed according to the intention-to-treat principle and addressed missing data for participant dropouts using the baseline-observation-carried-forward method to address missing data for participant dropouts. The Shapiro–Wilk test was used to examine the normality of the data. Analysis of covariance (ANCOVA) was employed to compare the improvement (post-intervention compared with pre-intervention) in clinical assessment scores and spatiotemporal symmetry ratios between the groups, with baseline values used as covariates. If significant effects were observed in the ANCOVA analysis, *post hoc* comparisons were conducted between the groups using the Wilcoxon signed-rank test for ordinal variables and the independent samples t-test for continuous variables. Furthermore, paired *t*-test was used to compare varies between pre-and post-intervention.

Additional simple linear regression analyses were performed to explore the correlation between the SRE-induced changes in spatiotemporal symmetry ratios and those in primary clinical outcomes (i.e., self-selected speed during the 10MWT and distance walked during the 6MWT) at post-intervention compared with pre-intervention.

In addition to statistical analyses, comparisons between changes in clinical outcomes and minimal clinically important differences (MCID) were performed to assess the clinical significance of changes in primary clinical outcomes. Based on previous acute-stroke studies, the MCID in 10MWT and 6MWT from pre-to post-intervention was determined to be 0.14 m/s (33) and 34.4 m (34), respectively. In all statistical analyses, significance was set at α = 0.05.

## 3. Results

### 3.1. Patient characteristics and clinical outcome measures

**Figure 2:**
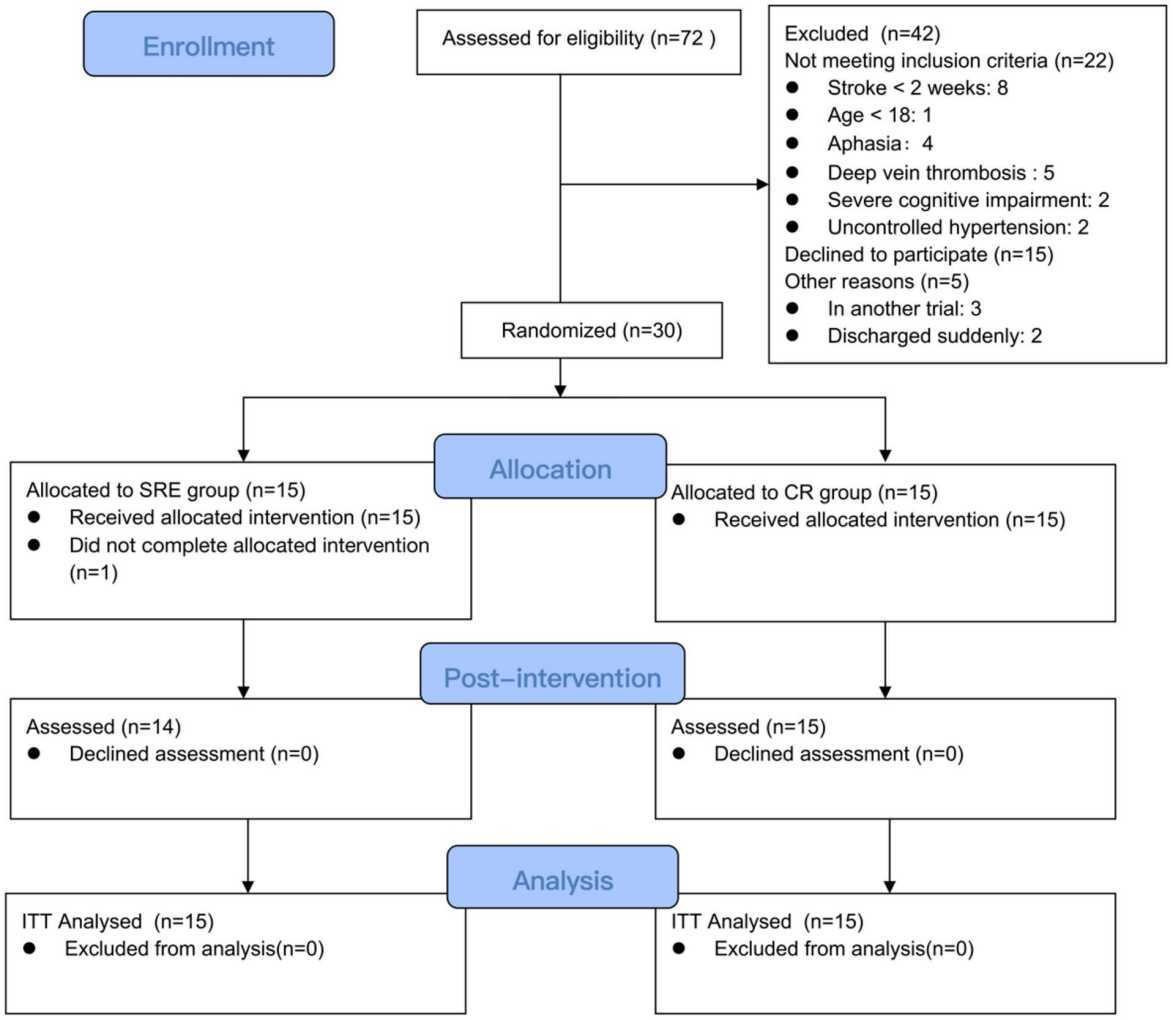
CONSORT flowchart of enrollment of participants into the study.

Overall, 72 patients with subacute stroke were screened for enrollment. Of these, 42 were excluded based on the exclusion criteria. Then, the 30 eligible individuals were randomized to the SRE group (*n* = 15) or CR group (*n* = 15). One participant dropped out of the SRE group after baseline testing because of anxiety and fear during SRE use. No significant between-group differences in the demographic characteristics (Table 1) or baseline clinical assessment scores (Table 2) were observed. Neither group had any serious adverse events while receiving intervention. The patient flowchart is shown in Figure 2.

**Table 1:** Demographic characteristics.

Characteristics	SRE group (*n* = 15)	CR group (*n* = 15)	*p*-value
Age, year, mean (SD)	59.00 (8.13)	63.30 (6.87)	0.056
Sex, male, *n* (%)	12 (80%)	9 (60%)	0.473
Body mass index, mean (SD)	25.07 (1.95)	24.59 (2.84)	0.646
Time since stroke, weeks, mean (SD)	8.27 (4.51)	8.53 (4.19)	0.764
Side of paresis, right, *n* (%)	10 (67%)	10 (67%)	0.400
Type of stroke, ischemic/hemorrhagic, n	10/5	13/2	0.294

SD, standard deviation; SRE, Soft Robotic Exoskeleton device; CR, conventional rehabilitation.

**Table 2:** Clinical outcome measures at baseline (pre) and within-group differences after intervention (post–pre).

Variables	SRE group (*n* = 15)	CR group (*n* = 15)
10MWT, m/s		
Pre	0.47 (0.17)	0.49 (0.18)
Post–pre	0.15 (0.11–0.19)^***^	0.09 (0.06–0.12)^***^
% Post-pre>MCID (0.05 m/s)	60%	33%
6MWT, m		
Pre	155.02 (48.66)	160.36 (55.38)
Post–pre	35.70 (26.02–45.38)^***^	24.26 (14.93–33.59)^***^
% Post-pre>MCID (34.3 m)	47%	20%
FAC		
Pre	3.47 (0.52)	3.40 (0.63)
Post–pre	0.33 (0.06–0.6)^*^	0.27 (0.01–0.52)^*^
% FAC ≥ 4 (independent walker)	73%	53%
FMA-LE		
Pre	20.47 (1.92)	20.73 (2.12)
Post–pre	4.13(2.66–5.61)^***^	2.27 (0.94–3.60)^**^
BBS		
Pre	40.40 (7.97)	40.53 (7.48)
Post–pre	2.60 (1.09–4.11)^**^	2.67 (0.82–4.51)^**^
Assistive level (N)		
Mean across 10 sessions	145.55 (14.6)	

For the SRE-assisted training, the assistive torque level and training intensity in terms of the mean step covered across 10 sessions are presented as mean (SD). The clinical outcomes of FAC scores are presented as medians (interquartile ranges). Pre values are presented as mean (SD), whereas post–pre values are presented as mean/median difference (95% CI). 10MWT, 10-Meter Walk Test; 6MWT, 6-Minute Walk Test; FAC, Functional ambulation category; FMA-LE, Fugl-Meyer Assessment for Lower Extremity subscale; BBS, Berg Balance Scale. ^*^p < 0.05, ^**^p < 0.01, ^***^p < 0.001, significant within-group difference.

In the intention-to-treat analysis of primary clinical outcomes, both groups showed significantly increased self-selected 1walking speed as measured by the 10MWT, and walking distances as measured by the 6MWT, across 10 training sessions (*p* < 0.001). However, after adjusting for baseline (pre-intervention) scores as covariates, ANCOVA results revealed that the SRE group achieved significantly greater improvement in the self-selected walking speed (adjusted group difference: +0.05 m/s, 95% confidence interval [CI] [0.02–0.09]; *F* = 8.675, *p* = 0.007) and walking distance (adjusted group difference: +10.07 m, 95% CI [1.98–18.15]; *F* = 6.525, *p* = 0.017) than those of the CR group at post-intervention compared with pre-intervention (Table 3).

**Table 3:** Adjusted between-group differences in clinical and symmetrical outcome improvements (post–pre), using baseline values as covariates.

Outcome measures	SRE group vs. CR group
10MWT, m/s	+0.05 (0.02–0.09) ** [0.243]
6MWT, m	+10.07 (1.98–18.15) * [0.195]
FAC	−0.10 (−0.33 to 0.13) [0.003]
FMA-LE	+1.68 (0.07–3.29) * [0.145]
BBS	−0.10 (−2.00 to 1.81) [0.011]
TSR	−0.26 (−0.42 to −0.11) ** [0.310]
SSR	−0.06 (−0.09 to −0.03) *** [0.437]

All analyses of covariance between-group differences were presented as mean difference (95% CI) [effect size: η2]; ^*^p < 0.05, ^**^ *p* < 0.01, ^***^p < 0.001, significant between-group differences.

The secondary clinical outcomes, measures using the FAC, FMA-LE, and BBS scores, are presented in Table 2. Both groups exhibited significant within-group differences (all *p* < 0.05) in the FAC, FMA-LE, and BBS scores across the 10-session training periods, indicating that both groups showed significant improvements in walking independence, motor recovery, and balance at post-intervention compared with pre-intervention. However, there were no significant between-group differences in these clinical scores (Table 3), except for the FMA-LE scores. The SRE group showed significant increases in the FMA-LE scores [adjusted group difference: +1.68, 95% CI (0.07–3.29); *F* = 7.555, *p* = 0.011] than the CR group at post-intervention compared with pre-intervention.

The clinical significance of the intervention was then investigated by comparing the primary clinical outcome measures (10MWT and 6MWT) with the MCIDs. The average improvements in 10MWT self-selected walking speed and 6MWT walking distance in the SRE group at post-intervention compared with pre-intervention were 0.15 ± 0.08 m/s and 35.70 ± 17.47 m, respectively, which were greater than the established MCIDs of the 10MWT (0.14 m/s) and 6MWT (34.4 m), respectively. In contrast, the average improvements in these clinical scores in the CR group at post-intervention compared with pre-intervention were 0.09 ± 0.05 m/s and 24.26 ± 16.85 m, respectively, and did not exceed the determined MCIDs of the 10MWT and 6MWT, respectively (Table 2).

### 3.2. Gait quality outcome measures

To investigate the mechanism through which interventions influenced gait performance, spatiotemporal and symmetry parameters during walking at two points (pre-and post-intervention) were collected and analyzed. Regarding spatiotemporal characteristics, the SRE group showed significant increases in step length on both the affected and unaffected sides (all *p* < 0.01) at post-intervention compared with pre-intervention, with no significant changes in other spatiotemporal parameters in either group (Table 4).

**Table 4:** Gait spatiotemporal characteristics at baseline (pre) and within-group differences after intervention (post–pre).

Gait parameters	Affected side		Unaffected side	
	SRE group	CR group	SRE group	CR group
	(*n* = 14)	(*n* = 15)	(*n* = 14)	(*n* = 15)
Step length, m				
Pre	0.70 (0.16)	0.70 (0.15)	0.73 (0.20)	0.74 (0.15)
Post–pre	0.08 (0.03–0.12)^**^	0.02 (−0.01 to 0.04)	0.04 (0.01–0.07)^**^	0.01 (0–0.02)
Step stance time, s				
Pre	1.14 (0.12)	1.18 (0.14)	1.35 (0.21)	1.29 (0.24)
Post–pre	0.09 (−0.04 to 0.21)	0.01 (−0.20 to 0.19)	−0.05 (−0.17 to 0.07)	−0.02 (−0.19 to 0.17)
Step swing time, s				
Pre	0.53 (0.10)	0.52 (0.09)	0.42 (0.10)	0.43 (0.10)
Post–pre	−0.02 (−0.05 to 0.00)	−0.01 (−0.05 to 0.02)	0.03 (−0.01 to 0.08)	0.01 (−0.01 to 0.03)

Pre values are presented as mean (SD), whereas post–pre values are presented as mean differences (95% CI). ^*^p < 0.05, ^**^p < 0.01, ^***^p < 0.001, significant within-group difference.

Changes in the symmetry ratios of the spatiotemporal parameters at two points were also examined. Table 5 presents the changes in TSR and SSR at two points. The SRE group showed significant reductions in both the TSR and SSR by-0.40 (t = −6.952, *p* < 0.001) from 1.66 ± 0.09 (pre-intervention) to 1.11 ± 0.29 (post-intervention) and by −0.08 (t = −3.664, *p* < 0.01) from 1.07 ± 0.07 (pre-intervention) to 1.02 ± 0.02 (post-intervention), respectively, whereas no significant changes were observed in the CR group. In addition, the ANCOVA results, using baseline values as covariates, revealed that the SRE group had significantly greater reductions in the SSR (adjusted group difference: -0.26, 95% CI [−0.42 to 0.11]; *F* = 12.136, *p* = 0.002) and TSR (adjusted group difference: -0.06, 95% CI [−0.09 to 0.03]; *F* = 20.955, *p* < 0.001) than the CR group at post-intervention compared with pre-intervention. Overall, the SRE group exhibited significantly greater reductions in both temporal and spatial symmetry ratios (indicating better gait symmetry) during walking than the CR group.

**Table 5:** Spatiotemporal symmetry parameters at baseline (pre) and within-group differences after intervention (post–pre).

Variables	SRE group (*n* = 15)	CR group (*n* = 15)
TSR, mean (SD)		
Pre	1.66 (0.09)	1.60 (0.12)
Post–pre	−0.40 (−0.52 to −0.27) ^***^	−0.11 (−0.28 to 0.06)
SSR, mean (SD)		
Pre	1.10 (0.09)	1.08 (0.05)
Post–pre	−0.08 (−0.13 to −0.03) ^**^	−0.01 (−0.02 to −0.01)

Pre values are presented as mean (SD), whereas post-pre values are presented as mean/median difference (95% CI). ^*^ *p* < 0.05, ^**^p < 0.01, ^***^p < 0.001, significant within-group difference.

**Figure 3:**
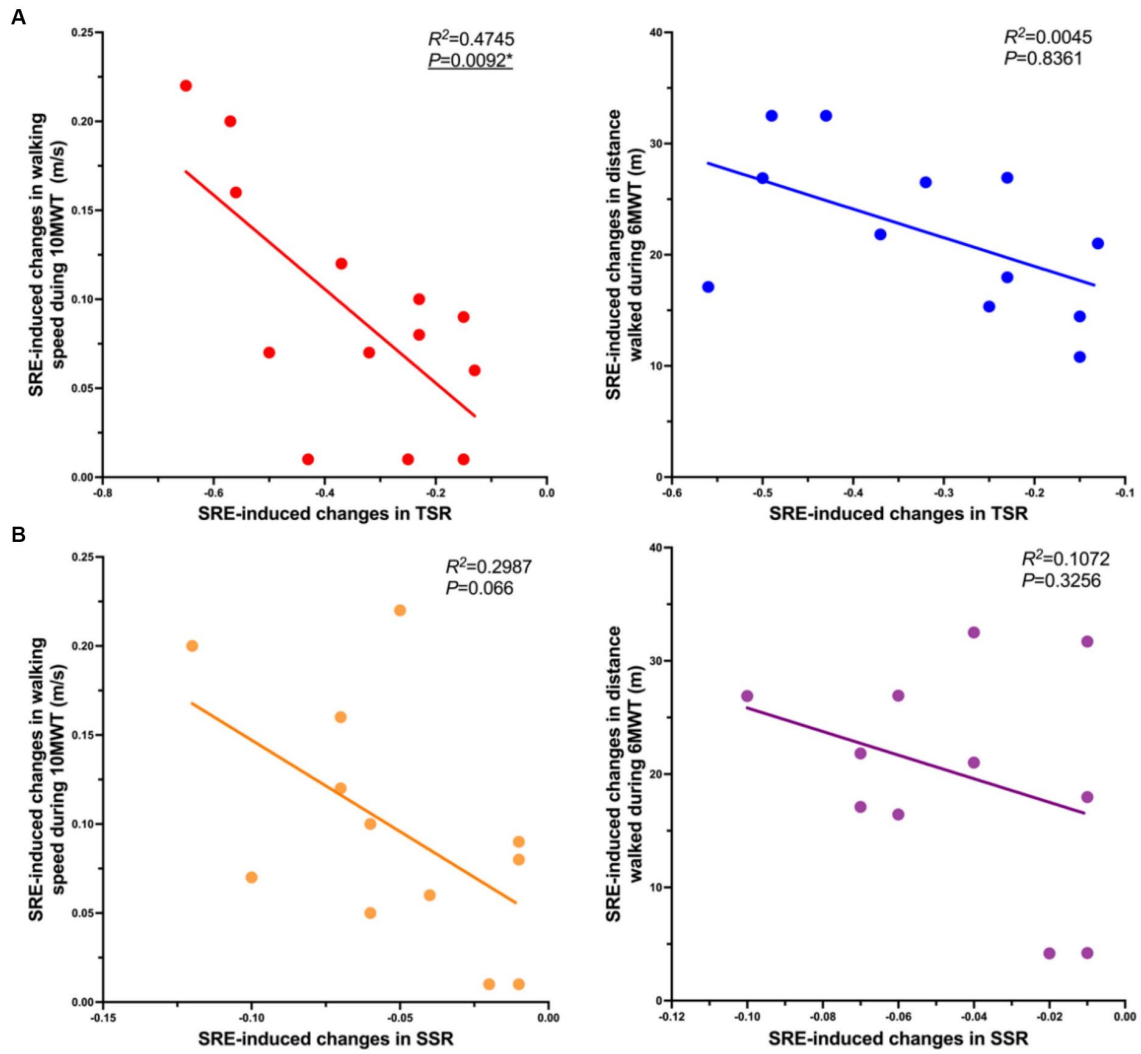
Correlations in SRE-induced changes in symmetry ratios and primary clinical outcomes, **(A)** Correlation of SRE-induced changes (Post-Pre) in TSR (horizontal axis, X) and SRE-induced changes in primary clinical outcomes (gait speed and walking distance) (horizontal axis, Y); **(B)** Correlation of SRE-induced changes in the SSR (horizontal axis, X) and the SRE-induced changes in primary clinical outcomes (gait speed and walking distance) (horizontal axis, Y). A simple linear regression analysis was performed after removing outliers of the X and Y axis parameters, and outliers were identified as greater than the mean ± 3 times the SD.

To further investigate the potential mechanisms underlying the SRE-induced changes in walking performance, additional correlation analyses using linear regression were performed to compare SRE-induced changes in spatiotemporal symmetry ratios and those in primary clinical outcomes (self-selected walking speed during the 10MWT and distance walked during the 6MWT). The results revealed a significant correlation between changes in the temporal symmetry ratio and those in walking speed (R^2^ = 0.475, *p* = 0.009), with no significant correlations between other variables (Figure 3). Notably, SRE-induced changes in both temporal and spatial symmetry ratios showed a negative correlation trend with changes in primary clinical outcomes.

## 4. Discussion

To the best of our knowledge, the present pilot study is the first RCT to explore the therapeutic effects of SRE when integrated within the CR protocol for patients who have experienced subacute stroke. The major findings were as follows. First, after a 10-session training, both groups demonstrated significant within-group improvements in all clinical outcome measures. Second, the SRE group showed greater improvements in walking speed (10MWT), walking endurance (6MWT), and motor recovery (FMA-LE) than the CR group. Third, gait analyses showed that the SRE group demonstrated a significant improvement in spatiotemporal gait symmetry after 10 training sessions, whereas no significant changes were observed in the CR group. This suggests that the recovery of walking function could be achieved by reducing gait asymmetry. The present suggests that the SRE can serve as a therapeutic tool to improve walking function during the subacute phase of stroke rehabilitation.

After 10 training sessions, the SRE and CR groups exhibited significant improvements in all clinical tests, including the 10MWT, 6MWT, FMA-LE, FAC, and BBS. Notably, we observed that walking speed (10MWT) and endurance (6MWT) were significantly improved in both groups after ten 30-min training sessions. The SRE and CR groups showed mean improvements of 0.15 ± 0.08 m/s and 0.09 ± 0.05 m/s in the 10MWT, and 35.70 ± 17.48 m and 24.26 ± 16.85 m in the 6MWT, respectively. However, between-group comparisons revealed that the SRE group outperformed the CR group in all the above clinical scores, and both 10MWT and 6MWT values were better than their MCIDs. This finding is consistent with that of a previous exploratory clinical study that included eight patients with chronic stroke who performed 18 SRE-assisted walk-training sessions for 520 h. Their result indicated a mean improvement of 0.22 ± 0.1 m/s in the 10MWT and 71.5 ± 43.9 m in the 6MWT (23). The previous study reported approximately 2-fold superior improvement in the 10MWT and 6MWT, compared with that of the present study; however, the reliability of the results of the previous was challenged by the self-repair effect of patients with stroke because no control group was included (35). The present study compensated for these shortcomings by including a control group, thereby providing more reliable results. The positive effects of SRE on walking speed and endurance may be attributed to the ability of the device to help patients to complete task-oriented, high-intensity, high-repetition early walking training by providing walking assistance. Previous studies have demonstrated that early task-oriented, high-intensity, high-repetition rehabilitation induces greater neuroplastic changes and functional recovery (36–38). In contrast, traditional rehabilitation training programs are limited by human and environmental constraints to achieve the training parameters advocated in appellate neuroplasticity theory (39).

The results of the present study were also comparable with those of another study evaluating a power-assisted ankle robot, which showed a significant improvement in clinical scores after 20 sessions (540 h) of robot-assisted walking training (40). In the present study, the SRE group showed improved 10MWT and FAC values after 10 training sessions, with improvements of 32% (0.15 m/s) and 10% (0.33 points), respectively. Similarly, using a power-assisted ankle robot, the patients exhibited 240% (0.32 m/s) and 74% (1.4 points) improvements in 10MWT and FAC, respectively, after 20 training sessions. The favorable improvements in 10MWT and FAC in this study were likely attributed to the presence of a ceiling effect from the included variables, as the baseline scores in the present study (0.47 m/s and 3.47 points for 10MWT and FAC, respectively) were significantly higher than those in the ankle robotic exoskeleton study (0.13 m/s and 1.9 points for 10MWT and FAC, respectively). Notably, the present study’s results showed no significant difference in the FAC score between the SRE and CR groups before and after the test; however, the SRE group had a higher proportion of patients who achieved the limited community independent walking (FAC ≥ 4) after 10 sessions of training than the CR group (73% vs. 53%) (27). This finding is consistent with that of the ankle robotic exoskeleton study, wherein approximately 57.1 and 29.4% of the ankle robotic exoskeleton and control groups, respectively, achieved limited community independent walking. These results suggest that ankle robotic exoskeleton-assisted walking training can improve patients’ walking independence. Nonetheless, the reliability of the results of the present study should be validated in larger populations for a more reasonable comparison given the limited sample size of the present study.

In addition to clinical scores, we measured the effects of the intervention on the spatiotemporal and symmetry parameters of the patients while walking. The results showed no significant changes in most spatiotemporal parameters between the SRE and CR groups after 10 training sessions; however, there were significant differences in the temporal and spatial symmetry ratios before and after training between the two groups. After 10 training sessions, the SRE group showed a significant decrease in both temporal and spatial symmetry ratios from a mean of 1.66 ± 0.09 and 1.10 ± 0.09, respectively, at baseline to a mean of 1.27 ± 0.17 and 1.02 ± 0.02, respectively, at the endpoint. In contrast, the CR group showed no significant changes in spatial and temporal symmetry ratios. These results suggest that compared with CR, SRE-assisted walking training effectively improves spatiotemporal symmetry during walking in patients with stroke, as a symmetry ratio closer to 1 indicates better motion symmetry between the limbs (32). This finding is similar to that of a previous lower-limb exoskeleton study, which found that four sessions of lower-limb exoskeleton robot-assisted flat-floor walking training significantly improved the spatial symmetry ratio of walking in patients with chronic stroke by approximately 13% (41). Another study on the energetics of SRE-assisted walking yielded consistent results, showing that SRE effectively improved the symmetry of the center of mass power of the body while walking in patients with hemiplegia, leading to a healthier and more efficient gait (21). According to the present study’s results, it is reasonable to suggest that SRE achieves a therapeutic effect on gait symmetry, which may also explain its potential mechanisms for improving walking performance and indirectly supports the conclusion that the positive effect of SRE on clinical scores is not achieved by reinforcing compensatory movement patterns. SRE-assisted walking training can induce plastic changes in the motor cortex through repetitive high-intensity proprioceptive stimulation and significant plantar pressure feedback, generating positive changes in gait patterns (17, 42, 43). In addition, neuroscience studies have shown that robotic gait training with voluntary drive and afferent feedback might lead to changes in the excitability of the motor cortex (27, 44). However, future studies are needed to further enhance our understanding of the role of soft robotic exoskeleton in neuroplasticity during walking recovery in patients with stroke.

To explore the underlying mechanisms of SRE in improving gait patterns, we examined the potential relationship between SRE-induced changes in the primary clinical scores (10MWT walking speed and 6MWT walking distance) and changes in gait symmetry (TSR and SSR) using simple linear regression analysis. The results showed a significant negative correlation between changes in the TSR and those in 10MWT walking speed, whereas no significant correlations were observed for the remaining data. Notably, both TSR and SSR showed a similar trend of negative correlation with walking speed and distance. This result implied that the greater the SRE-induced decrease in TSR and SSR (the closer to 1), the more optimal the 10MWT walking speed and 6MWT walking distance. This negative correlation trend was also validated by previous studies in which the degree of improvement in walking symmetry in patients with stroke had a linear relationship with improvements in walking propulsion, speed, and efficiency (32, 45, 46). Based on these findings, it is reasonable to speculate that the significant improvement in walking performance (indicated by clinical assessment scores) induced by the SRE resulted from improved gait symmetry. However, because this is only a preliminary conjecture based on the limited available data, additional high-quality research is required for further testing.

This study has several limitations. First, the sample size was small for an RCT, which reduced the generalizability of the findings and raised the possibility of type II errors. Because of the limited sample size, we could not account for data stratification or temporal correlation in the statistical analysis, which also raised the possibility of type II errors. However, this pilot study was designed to support subsequent large-scale RCT. Therefore, we will compensate for this limitation by conducting a subsequent RCT with a larger sample size. Second, there was no follow-up in this study; therefore, whether the therapeutic effects of SRE are long-lasting needs to be verified in further trials with long-term follow-up. Third, other than the robot-assisted walking training, the training intensity and content of the CR training sessions was not standardized in the research design, which may have resulted in bias in the CR group that underwent conventional training. Finally, data on lower-extremity joint kinematics and walking dynamics before and after training were not collected in this study. Therefore, the biomechanical effects of the SRE in patients with stroke need to be verified in future studies.

## 5. Conclusion

This RCT demonstrated the therapeutic efficacy of a 10-session SRE-assisted walking training for functional walking recovery in survivors of subacute stroke. SRE-assisted walking training led to greater improvements in walking speed, walking endurance, and motor recovery than did CR training. In addition, gait quality analyses showed that SRE-assisted walking training could improve spatiotemporal symmetry during walking, which may be a potential mechanism underlying SRE-induced improvement in walking performance. This study provides preliminary evidence that SRE may be considered for inclusion in intensive gait training clinical rehabilitation programs to further improve walking function in patients who have experienced stroke. These promising results required validation in future RCTs with larger sample sizes and long-term follow-up.

## Data availability statement

The raw data supporting the conclusions of this article will be made available by the authors, without undue reservation.

## Ethics statement

The studies involving humans were approved by the Ethics Committee of Beijing Tsinghua Changgung Hospital. The studies were conducted in accordance with the local legislation and institutional requirements. The participants provided their written informed consent to participate in this study.

## Author contributions

RX: Conceptualization, Data curation, Formal analysis, Funding acquisition, Investigation, Methodology, Project administration, Resources, Software, Supervision, Validation, Visualization, Writing – original draft, Writing – review & editing. YZ: Data curation, Formal analysis, Investigation, Resources, Writing – review & editing. HJ: Data curation, Formal analysis, Investigation, Resources, Writing – review & editing. FY: Conceptualization, Investigation, Methodology, Project administration, Supervision, Writing – review & editing. YF: Formal analysis, Investigation, Methodology, Supervision, Writing – review & editing. YP: Formal analysis, Investigation, Methodology, Supervision, Writing – review & editing, Conceptualization, Data curation, Funding acquisition, Project administration, Resources, Software, Validation, Visualization.
